# Effects of Ovariohysterectomy and Hyperbaric Oxygen Therapy on Systemic Inflammation and Oxidation in Dogs

**DOI:** 10.3389/fvets.2019.00506

**Published:** 2020-01-15

**Authors:** Anais Gautier, Emily C. Graff, Lenore Bacek, Eric J. Fish, Amelia White, Lee Palmer, Kendon Kuo

**Affiliations:** ^1^Department of Emergency and Critical Care, Auburn University Veterinary Teaching Hospital, Auburn, AL, United States; ^2^Department of Pathobiology, Auburn University Veterinary Teaching Hospital, Auburn, AL, United States; ^3^Department of Dermatology, Auburn University Veterinary Teaching Hospital, Auburn, AL, United States

**Keywords:** canine, hyperbaric oxygen therapy, ovariohysterectomy, inflammation, cytokines, oxidation, iron

## Abstract

**Introduction:** Hyperbaric oxygen therapy (HBOT) involves breathing 100% oxygen in a specialized compression chamber leading to hyperoxia. This treatment modality is associated with anti-inflammatory, antioxidant, and healing properties in people and laboratory animals. However, there are relatively few reports that evaluate the effects of HBOT in companion animals. The goal of this study was to investigate the physiological effects of HBOT on surgically induced systemic inflammation and oxidation in dogs.

**Material and Methods:** Twelve healthy female beagle dogs were spayed and randomized into control and HBOT groups (*n* = 6). Both groups received conventional post-ovariohysterectomy therapy, and the HBOT group received two hyperbaric treatments at 2.0 atmosphere of absolute pressure and 100% oxygen for 35 min, 6 and 18 h after surgery. Blood samples were collected 3 h prior to ovariohysterectomy, 6, 18, and 30 h after surgery, prior to HBOT when applicable. Inflammatory biomarkers, including C-reactive protein, circulating cytokines, and changes in iron homeostasis were evaluated at each time point to determine the effects of surgery and HBOT on inflammation. Similarly, serum total oxidant status and total antioxidant status were measured to assess the oxidative stress. Pain and incision scores were recorded and compared between groups.

**Results:** Following ovariohysterectomy, all dogs had significantly increased serum concentrations of C-reactive protein, KC-like, IL-6, and increased unsaturated iron-binding capacity compared to their pre-surgical values (*p* < 0.02), while serum iron, total iron-binding capacity and transferrin saturation were significantly decreased after surgery (*p* < 0.02). There was no significant difference between the control group and the HBOT group for any of the variables. There were no overt adverse effects in the HBOT group.

**Conclusion:** This is the first prospective randomized controlled study to investigate the effects of HBOT on surgically induced systemic inflammation in dogs. While elective ovariohysterectomy resulted in mild inflammation, the described HBOT protocol portrayed no outward adverse effect and did not induce any detectable pro-inflammatory, anti-inflammatory, or antioxidant effects. Additional investigation is required to identify objective markers to quantify the response to HBOT and determine its role as an adjunctive therapy in dogs with more severe, complicated or chronic diseases.

## Introduction

Hyperbaric oxygen therapy (HBOT) is a medical treatment modality gaining popularity in both human and veterinary medicine for its apparent acceleration of tissue healing (1–8). During treatment, the patient breathes 100% oxygen inside a pressurized chamber above normal, sea level, atmospheric pressure [1 atmosphere of absolute pressure (ATA)]. Under these conditions, based on ideal oxygen pressure laws and an oxygen solubility in blood of 0.0031 mL/dL of blood per mmHg of arterial oxygen tension, the partial pressure of alveolar oxygen greatly increases from 100 mmHg on room air at 1 ATA to 1,400 mmHg on 100% oxygen at 2 ATA leading to transient hyperoxia (9). Primary effects of HBOT are improved tissue oxygenation (9) and reduction of intravascular and tissue gas bubble in relation to Boyle's law (10). Secondary physiologic effects are the results of short-term sub-lethal oxidative stress and include compensatory antioxidant response (3, 11, 12), modulation of inflammation and immune function (4, 5, 13), increased antimicrobial activity (14, 15), angiogenesis (16), enhanced fibroblast activation (17), upregulation of growth factors (18), vasoconstriction, and reduction of vasogenic edema (19). Although rare, possible complications related to increased atmospheric pressure, increased partial pressure of alveolar oxygen and hyperoxia include pneumothorax and other forms of barotrauma, oxygen lung toxicity and seizures, respectively (20).

The current understanding of the physiology and mechanisms of action of HBOT results primarily from investigations in people and laboratory animal (21). Prospective randomized controlled studies investigating the HBOT-mediated cellular response to oxidative stress in companion animals are lacking.

The goal of this study was to investigate the physiological effects of HBOT on surgically induced systemic inflammation and oxidation in dogs by comparing the expression of inflammatory biomarkers, pro-inflammatory and anti-inflammatory cytokines, total oxidant status, total antioxidant status, and oxidative stress index before and after postoperative HBOT. We hypothesized that HBOT would be safe under the proposed conditions and that it has anti-inflammatory and antioxidant properties in dogs undergoing ovariohysterectomy.

## Materials and Methods

### Study Population

Twelve 11–12-month-old intact female purpose-bred beagle dogs were found to be healthy based on their medical history, physical examination, hematological, and biochemistry profiles, urine analysis, vaginal swab, ZnSO4/I2 fecal flotation and SNAP 4Dx® assay (IDEXX Laboratories, Westbrook, ME, USA). Exclusion criteria are listed in Table 1. All animals were housed in a facility accredited by the United States Department of Agriculture and the Association for Assessment and Accreditation of Laboratory Animal Care International for the duration of the study. The dogs were cared for in compliance with the Animal Welfare Acts and fed the same diet (Pro Plan Adult Chicken & Rice Formula, Nestle/Purina, St. Louis, MO, USA) based on their daily energy requirement. The study was approved by the University Institutional Animal Care and Use Committee.

**Table 1:** Exclusion criteria.

Concurrent diseases that may influence inflammatory and antioxidant biomarkers including allergy and hypersensitivity, heartworm disease, severe intestinal parasite infections or ectoparasites associated with clinical signs
Medication other than heartworm and flea/tick preventative drugs
Recent vaccination
Estrus within the 30 days preceding surgery
Pregnancy
Recent surgery < 1 month
Breaks in aseptic technique during surgery
Major post-operative complications: hemoabdomen, septic abdomen, wound dehiscence, abscess
Contraindications for HBOT: claustrophobia, pneumothorax, otitis, history of ear or thoracic surgery, dental cavities, nasal congestion

### Anesthesia, Surgery, and Post-operative Care

All dogs were pre-medicated with hydromorphone (0.1 mg/kg) and dexmedetomidine (200 mcg/m^2^) intra-muscularly, and a peripheral intravenous catheter was placed. Anesthesia was induced with midazolam (0.2 mg/kg) and ketamine (5 mg/kg) intravenously, and maintained with isoflurane in 100% oxygen. During anesthesia, the dogs received Lactated Ringer's Solution (Vetivex®, Dechra, Overland Park, KS, USA) at a constant rate (5 mL/kg/h) intravenously. Intraoperative monitoring consisted of electrocardiography, capnography, body temperature, pulse oximetry (SurgiVet Advisor® 3 parameter vital signs monitor, Smiths Medical Inc., Minneapolis, MI, USA) and Doppler blood pressure (Doppler ultrasound, Parks Medical Electronics Inc., Aloha, OR, USA).

Ovariohysterectomies (OVE) were performed via ventral midline laparotomy by a single board-certified surgeon using a standardized surgical protocol. The length of the abdominal incision was consistent at ~13 cm. Duration of surgery, estimated blood loss, doses and time of drugs administered were recorded. After surgery, the dexmedetomidine was antagonized with 0.5 μg/m^2^ atipamezole administered intramuscularly.

Mentation was monitored hourly and a physical examination was performed every 6 h for the first 30 h, and once daily for 13 additional days. Post-operative care included administration of hydromorphone (0.05 mg/kg) intravenously every 6 h for the first 18 h. After collection of the final blood samples, the dogs received carprofen (2.2 mg/kg) by mouth every 12 h for 4 days for analgesia. Incision sites were uncovered, and the sutures were removed 14 days after surgery.

### Hyperbaric Oxygen Therapy

**Figure 1:**
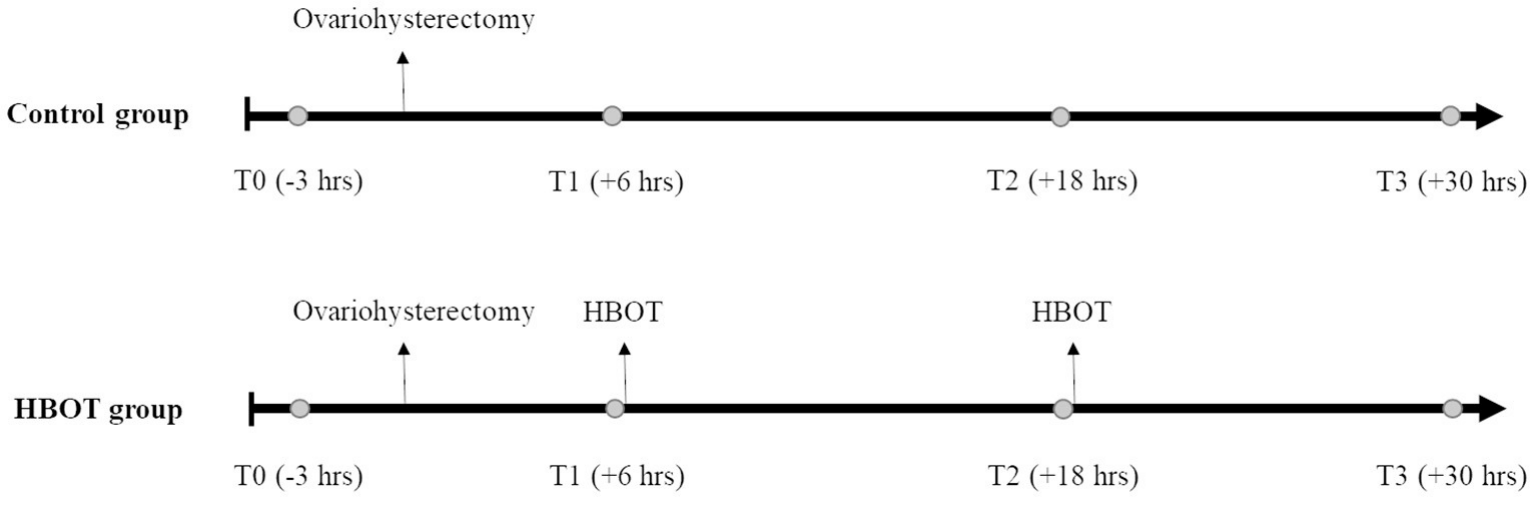
Experiment timeline. Whole blood (circle) is collected 3 h prior to ovariohysterectomy (T0), 6, 18, and 30 h after ovariohysterectomy (T1, T2, and T3, respectively), and prior to hyperbaric oxygen therapy (HBOT) for the HBOT group.

The dogs were randomly divided into a control group (control group) and HBOT group using a random integer set generator (www.random.org). The HBOT group received 2 hyperbaric treatments, 6 and 18 h after surgery (Figure 1). During hyperbaric treatments, the dogs were administered 100% oxygen at 2.0 ATA for 35 min in a purpose-built veterinary hyperbaric chamber (Hvm model 3200 veterinary hyperbaric chamber, Hyperbaric veterinary medicine®, Boca Raton, FL, USA), with a compression and decompression time of 10 min each. A veterinarian who completed an introductory course approved by the National Board of Diving and Hyperbaric Medical Technology (Hyperbaric medicine team training for animal applications, International ATMO Inc., San Antonio, TX, USA) supervised all hyperbaric treatments. Treatments were performed at least 4 h after the last administration of hydromorphone to decrease the risk for respiratory depression. Through the windows of the chamber, the dogs were monitored for inappropriate mentation, respiratory distress, seizures, signs of barotrauma (head shaking, discomfort, vocalization), and confinement anxiety. A full physical examination was performed immediately prior and after completion of the treatments.

### Clinical Outcomes

Post-operative wound healing and pain were evaluated by two non-blinded observers every 6 h for the first 30 h, and once daily for 13 additional days using the incision scoring system described by (22), and the pain scoring system developed by (23), respectively (22, 23).

### Inflammatory and Oxidative Biomarkers

Five milliliters of whole blood were collected by direct venipuncture of a jugular vein 3 h prior to surgery (T0) and then 6 h (T1), 18 h (T2), and 30 h (T3) after surgery (Figure 1). In the HBOT group, samples were collected prior to the hyperbaric treatments. Blood was allowed to clot for 30 min and centrifuged at 3,000 rpm for 9 min. All serum samples were stored at −80°C until use. Inflammatory biomarkers and oxidative stress were measured at all time-points, in a blinded fashion and in duplicate.

Serum cytokines, chemokines and growth factors concentrations were measured using commercially available enzyme-linked immunosorbent assays and Luminex multiplex technology. Milliplex MAP Canine Cytokine/Chemokine Magnetic Bead Panels (EMD Millipore Corporation, Burlington, MA, USA) were used to measure the following cytokines: interleukin (IL)-2, IL-6, IL-7, IL-8, IL-10, IL-15, IL-18, interferon γ (IFN-γ), interferon-γ-induced protein (IP-10), tumor necrosis factor alpha (TNF-α), monocyte chemoattractant protein 1 (MCP-1), granulocyte-macrophage colony-stimulating factor (GM-CSF), and keratinocyte chemotactic-like (KC-like). The results were read with a Luminex MAP 96-well-microplate reader (Luminex Corporation, Austin, TX, USA) at 511 nm and the data were analyzed using xPONENT® (Luminex Corporation, Austin, TX, USA).

Serum samples were sent to the University of Miami Acute Phase Protein Laboratory for serum CRP levels measurements. Randox immunoturbidimetric Canine CRP assays (Randox, Kearneysville, WV, USA) were ran with a Daytona Rx analyzer (Randox, Kearneysville, WV, USA).

Serum iron (S. iron) and unsaturated iron-binding capacity (UIBC) were measured using the colorimetric Ferrozine method with the Roche Cobas 311 chemistry analyzer (Roche/Hitachi Ltd., Indianapolis, IN, USA). Total iron binding capacity (TIBC) and transferrin saturation (TSAT) were calculated by the following:

$$\begin{array}{l} \text{ TIBC}=\text{S. iron }+\text{ UIBC}\\ \text{TSAT}\%=\left[\text{S. iron}/\text{TIBC}\right]\;\times \;100 \end{array}$$

Serum total oxidant status (TOS) and total antioxidant status (TAS) levels were determined by the colorimetric methods described by Erel et al. in 2004 and 2005 using commercially available kits (Total oxidant status and total antioxidant status assay kits, Rel Assay Diagnostics, Gaziantep, Turkey) (24, 25). Oxidative stress index (OSI) was calculated by the following:

$$\begin{array}{l} \text{OSI}=\left[\left(\text{TOS},\mu \text{mol}/\text{L}\right)/\left(\text{TAS},\text{mmol Trolox equivalent}/\text{L}\right)\right]\times \;100 \end{array}$$

### Icteric Index, Hemolytic Index, Lipemic Index, Progesterone Concentration

Icteric, hemolytic and lipemic indices were measured by spectrophotometry in all samples using a Roche Cobas 311 chemistry analyzer (Roche/Hitachi Ltd., Indianapolis, IN, USA). Serum progesterone levels were measured at T0 using an enzyme-labeled chemiluminescent technology and Immulite 1000 (Siemens Healthcare Diagnostics Inc., Tarrytown, NY, USA).

### Statistical Analysis

All analyses were performed using a commercially available software SAS V 9.4 (SAS Institute Inc., Cary, NC, USA). Unpaired Student *t*-tests were used to identify differences in body weight and time of the surgical procedure between groups. Cytokine concentrations below the limit of detection were substituted with the limit of detection divided by the square root of 2 (26). For all inflammatory and oxidative stress biomarkers, outliers were identified prior to surgery at T0 using the median absolute deviation and a threshold of 3.5 (27). This allowed the identification of two dogs in the control group with pre-existing abnormal cytokine profile. No underlying cause was identified, and the dogs were excluded from the cytokine statistical analysis.

Model residuals were examined to evaluate the assumption of normality for all continuous data. Considering all serum concentrations were normally distributed, a linear mixed model was used to analyze the biomarker concentrations with inclusion of fixed factors such as group, time, and group by time interaction for each biomarker. Alternatively, to control for baseline differences and therefore possible differences in relative change, baseline biomarker concentration was added as a covariate. Lipemic index (at each time), hemolytic index (at each time) and progesterone (at first time) were initially included as fixed covariates for each biomarker, and backwards elimination allowed the identification of significant covariates. A random intercept for each dog was included in the mixed model to account for within dog repeated measures correlation over time. Satterthwaite degrees of freedom method allowed the comparison of means from two normal distributions with small samples and possibly different variances. All pairwise comparisons were corrected for multiple comparisons with Tukey's method. A *p*-value of < 0.05 was defined as the significance cut-off. Results are presented as mean ± standard deviation.

## Results

### Study Population and Clinical Outcome

The dogs' mean body condition score was 4.6/9, with no statistical difference between the control and HBOT groups. The control dogs weighed significantly more than the dogs treated with HBOT with a body weight of 10.3 (±0.462) kg, and 8.98 (±1.01) kg, respectively. This difference was statistically significant (*p* = 0.017) with no influence detected on other variables. Nine dogs were in anestrus and 3 dogs were more than 30 days into diestrus by the time of surgery. Among the 3 dogs in diestrus, 1 control dog and 1 HBOT dog had elevated blood progesterone level at T0 (25 and 7.05 ng/mL, respectively). The range of surgical times was 27–40 min, with a median time of 30.5 min and no significant difference between groups. There were no surgical complications observed. All dogs in the HBOT group completed the hyperbaric oxygen sessions without any observed adverse effects. Physical examinations were unremarkable before and after hyperbaric treatments. No dog required rescue analgesia during hospitalization in any of the groups. Pain and incision scores did not differ at any time between the control and HBOT groups. All incisions were healed without complication 14 days post-surgery by the time of suture removal, and all dogs were determined clinically healthy 2 months after the study.

Significant covariates for each biomarker are shown in Table 2.

**Table 2:** Slopes and *p*-values for significant covariates.

**Covariate**	**Biomarker**	**Slope**	***p*-value**
Hemolytic index	IL-2	0.26	0.0136
	IL-6	−0.71	0.0252
	IL-15	0.81	0.0044
	IL-18	0.23	0.0002
	GM-CSF	0.46	0.0340
	Serum iron	1.02	0.0021
	TIBC	0.43	0.0366
	TSAT	0.18	0.0232
Lipemic index	Serum iron	−4.5	0.0311
	TSAT	−1.1	0.0324
	UIBC	5.8	0.0098
	OSI	2.8	0.0449
Progesterone	IL-8	110	0.0068
	Serum iron	3	0.0218
	TSAT	0.58	0.0399
	TOS	0.012	0.0031

*IL, interleukin; GM-CSF, granulocyte-macrophage colony-stimulating factor; TIBC, total iron binding capacity; TSAT, transferrin saturation; UIBC, unsaturated iron-binding capacity; TOS, total oxidant status; OSI, oxidative stress index. For example, IL-2 increases with increasing hemolytic index (by an average of 0.26 units per 1 unit of hemolytic index, p = 0.0136)*.

### Acute Phase Protein and Iron Status

**Figure 2:**
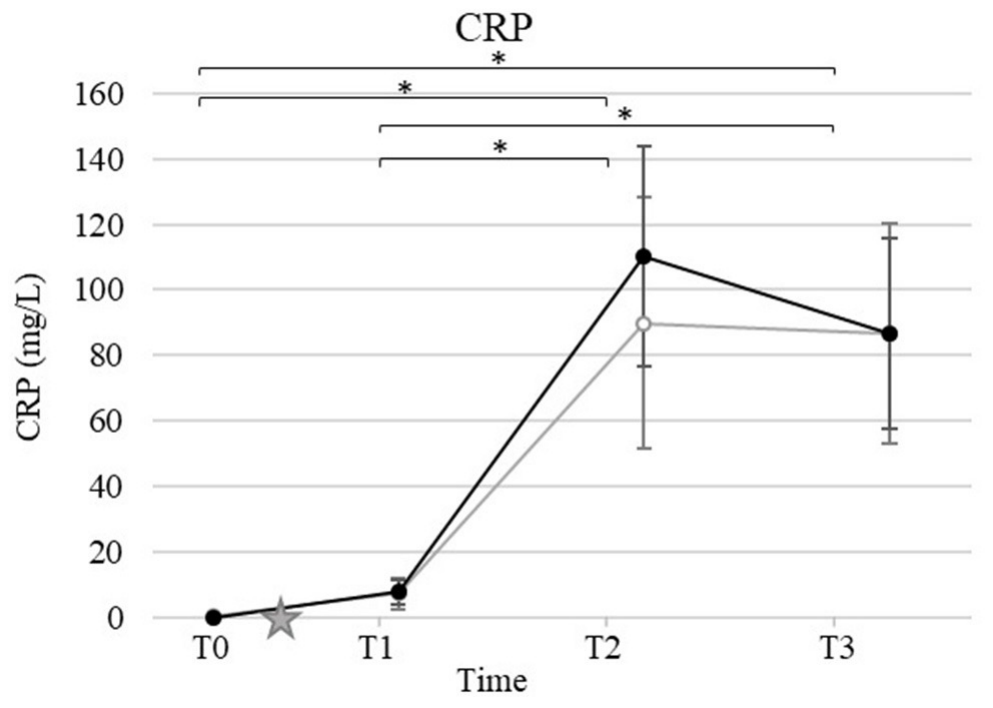
Mean and standard deviation of serum C-reactive protein (CRP) concentrations prior to ovariohysterectomy (T0), 6, 18, and 30 h after ovariohysterectomy (T1, T2, and T3, respectively) for the control group (gray line) and the hyperbaric oxygen therapy (HBOT) group (black line). The star indicates the time of surgery. *indicates a significant difference (*p* < 0.05) with time in both control and HBOT groups.

**Figure 3:**
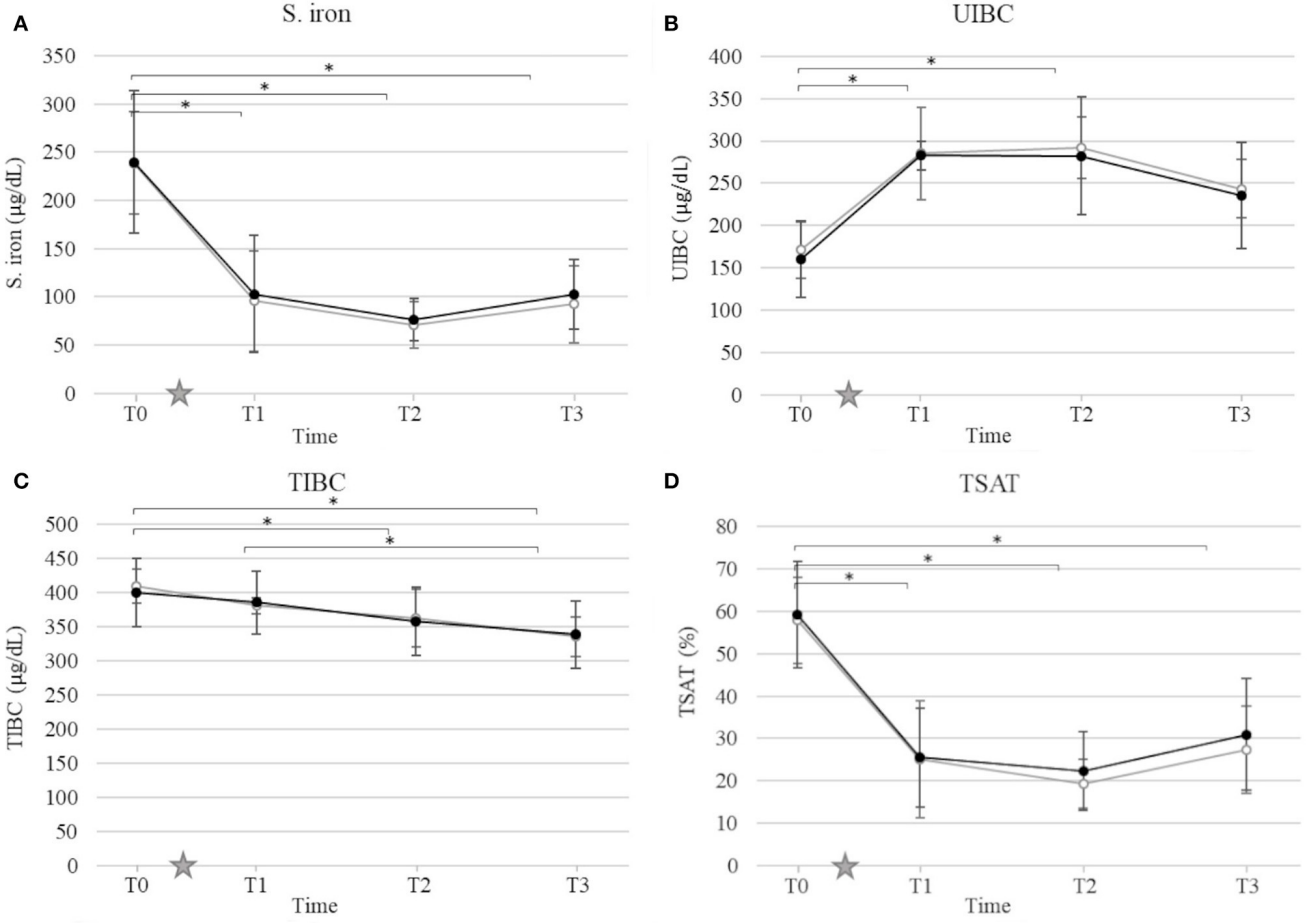
Mean and standard deviation of serum iron concentration (S. iron) **(A)**, unsaturated iron-binding capacity (UIBC) **(B)**, total iron-binding capacity (TIBC) **(C)**, and transferrin saturation (TSAT) **(D)** prior to ovariohysterectomy (T0), 6, 18, and 30 h after ovariohysterectomy (T1, T2, and T3, respectively) for the control group (gray line) and the hyperbaric oxygen therapy (HBOT) group (black line). The star indicates the time of surgery. *indicates a significant difference (*p* < 0.05) with time in both control and HBOT groups.

Mean and standard deviation for CRP, S. iron, UIBC, TIBC, and TSAT by time and group are presented in Figures 2, 3. Ovariohysterectomy induced a significant increase in CRP concentration at T2 and T3 (*p* < 0.01), and UIBC at T1 and T2 (*p* < 0.01) in both control and HBOT groups. Ovariohysterectomy induced a significant decrease in S. iron concentrations and TSAT at T1, T2, and T3 (*p* < 0.01), and TIBC at T2 and T3 (*p* = 0.01 and *p* < 0.01, respectively in the control group, and *p* = 0.04 and *p* < 0.01, respectively in the HBOT group). Total iron binding capacity was significantly increased at T3 compared to T1 in the HBOT group (*p* = 0.01) with no significant difference between control and HBOT groups. There was no effect of HBOT for any of the variables at any time point.

### Cytokines Profile

**Figure 4:**
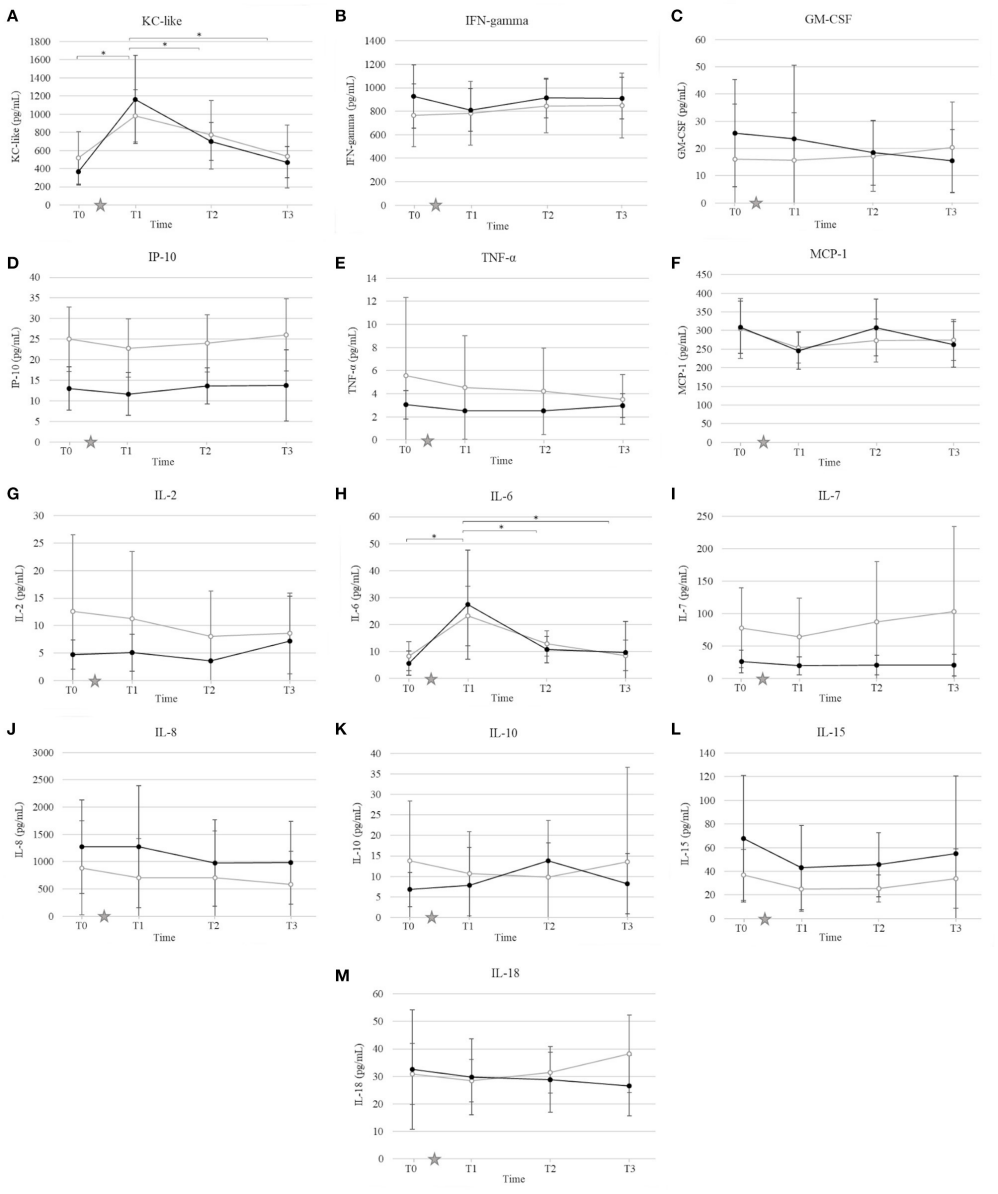
Mean and standard deviation of serum concentrations of keratinocyte chemotactic-like (KC-like) **(A)**, interferon γ (IFN-γ) **(B)**, granulocyte-macrophage colony-stimulating factor (GM-CSF) **(C)**, interferon-γ-induced protein (IP-10) **(D)**, tumor necrosis factor alpha (TNF-α) **(E)**, monocyte chemoattractant protein 1 (MCP-1) **(F)**, interleukin (IL) IL-2 **(G)**, IL-6 **(H)**, IL-7 **(I)**, IL-8 **(J)**, IL-10 **(K)**, IL-15 **(L)**, IL-18 **(M)** prior to ovariohysterectomy (T0), 6, 18, and 30 h after ovariohysterectomy (T1, T2, and T3, respectively) for the control group (gray line) and the hyperbaric oxygen therapy (HBOT) group (black line). The star indicates the time of surgery. *indicates a significant difference (*p* < 0.05) with time in both control and HBOT groups.

Ovariohysterectomy induced a ~4-fold increase in the serum concentration of KC-like in both control and HBOT groups at T1 (*p* < 0.01), and a ~2-fold increase in the IL-6 serum concentration in the HBOT group at T1 (*p* < 0.01) with no significant difference between control and HBOT groups (Figure 4). There was no significant effect of either OHE or HBOT on the remaining 11 cytokines.

### Oxidation Status

**Figure 5:**
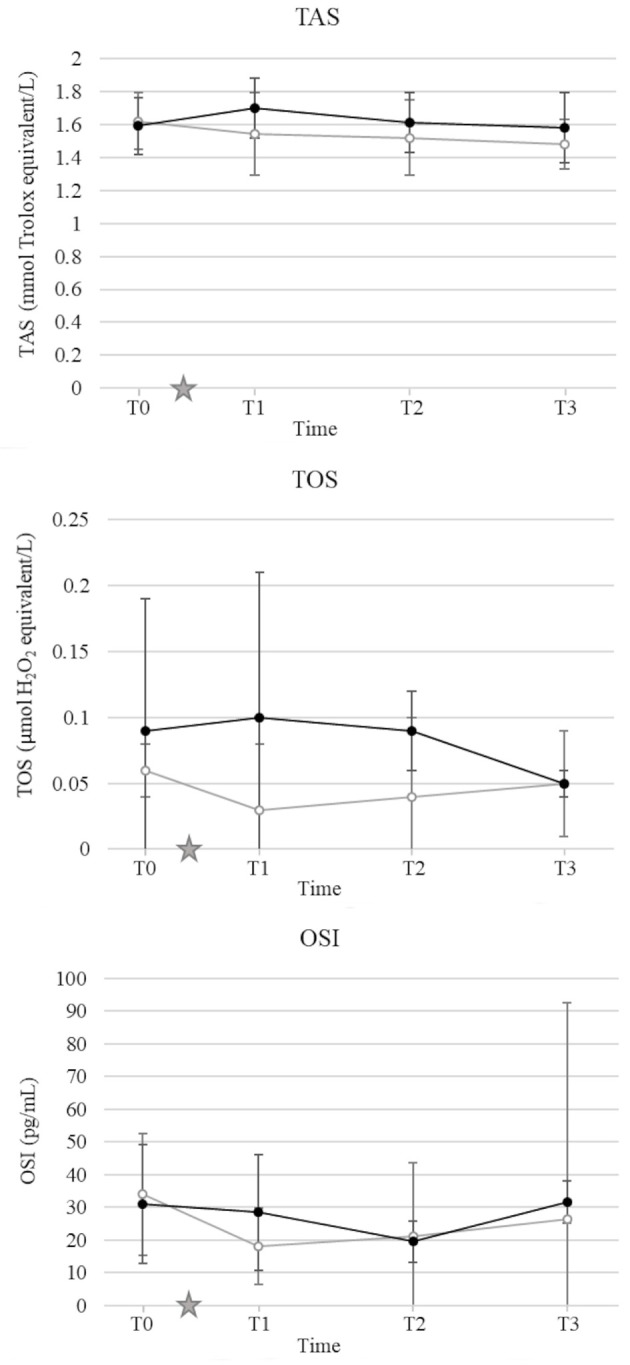
Mean and standard deviation of serum total antioxidant status (TAS) **(A)**, total oxidant status (TOS) **(B)**, oxidative stress index (OSI) **(C)** prior to ovariohysterectomy (T0), 6, 18, and 30 h after ovariohysterectomy (T1, T2, and T3, respectively) for the control group (gray line) and the hyperbaric oxygen therapy (HBOT) group (black line). The star indicates the time of surgery.

There was no significant effect of either OHE or HBOT on TOS, TAS and OSI (Figure 5).

## Discussion

Ovariohysterectomy induced a mild inflammatory response in dogs with no change detected in oxidative status at any sampling time. HBOT did not affect any of the variables. There was no difference detected between the control group and the HBOT group for any of the variables, suggesting no benefit nor harm of this HBOT protocol in dogs following OVE. HBOT appeared to be well-tolerated and portrayed no outward adverse effects at the dosage and duration used in the present study.

Since HBO is typically utilized as an adjunctive therapy in veterinary medicine, designing a study to evaluate its therapeutic effects with adequate power and minimal bias remains challenging. Ideally, HBOT would be the only treatment administered to a large population affected by a disease comparable in severity and measurable with specific and sensitive biomarkers, and comparable to a placebo. While these conditions are sometimes met with experimental laboratory animal models (28), they are difficult to achieve in a clinical setting. Additionally, most diseases that are reported to have potentially benefited from HBOT have varying degrees of severity with no disease specific biomarkers identified (i.e., pancreatitis, brain injury, sepsis, lung injury, wounds, and cardiopulmonary bypass surgery) (3–8, 13, 29, 30). For this study, an elective OHE performed by a skilled surgeon induced a mild inflammatory response as indicated by a short, but significant increase in CRP, UIBC, IL-6, and KC-like cytokine with a concurrent decrease in S. iron, TSAT, and TIBC in all dogs. Ovariohysterectomy is performed in a controlled environment with relatively comparable amounts of stress and tissue injury between patients, and limited medical intervention outside of analgesics. In order to limit the variability of inflammation induced in this study, a single surgeon performed the procedure using the same anesthetic and surgical protocols. Additionally, non-steroidal anti-inflammatory drugs were not initiated until after the last sampling time. Our results showed no statistical difference in biomarker concentrations, incision score, and pain score between groups prior to surgery, and prior to HBOT. Therefore, the clinical outcome, inflammation and oxidative stress induced by surgery were comparable immediately after OHE, and we speculated that differences between groups would be attributable to HBOT.

Interestingly, progesterone was identified as a significant covariate for IL-8, S. iron, TSAT, and TOS in this study. While it is known that the canine corpus luteum expresses various cytokines including IL-8 throughout diestrus (31), few data are available regarding the relationship between iron homeostasis, oxidative stress, and estrous phases in dogs. Studies with more dogs in different phases of the estrous cycle are warranted for further investigation. All dogs displayed a significant increase in CRP 18 h after surgery. These results are consistent with previous studies reporting an increase in CRP within the first 24 h after OHE with a subsequent reduction in the absence of ongoing trauma (32–36). HBOT has been shown, previously, to reduce CRP concentration in people with traumatic brain injury treated with 4 hyperbaric oxygen treatments at 3-day intervals (1 h on 100% oxygen at 2.0 ATA) (5), and in children with autism after 40 hyperbaric oxygen treatments at 1- to 2-day intervals (45 min on 100% oxygen at 1.3 and 1.5 ATA) (37). In our study, HBOT did not affect the concentration of CRP. This finding may indicate a lack of effect of HBOT in dogs at the lower dose used in this study, namely 2 treatments at 12-h interval for 35 min on 100% oxygen at 2.0 ATA. The small population and mild inflammation induced could also lead to a type II error, resulting in the absence of difference detected in CRP concentration between the control and HBOT groups. Further studies involving greater inflammatory stimuli and/or chronic inflammation (i.e., orthopedic surgery, pancreatitis, dermatitis), exposure to more and/or longer hyperbaric oxygen treatments, and measurements of CRP over a longer period are needed before determining the lack of effects of HBOT on inflammatory acute phase proteins in dogs.

Hypoferremia is a sensitive marker of systemic inflammation, including surgically induced inflammation (36, 38–41). Changes in iron homeostasis occur within the first 24 h after surgery, progressively normalize as inflammation subsides (36, 41), correlate with the extent of surgery (38), and serve as a valuable biomarker to monitor systemic inflammation and predict clinical outcome (39, 40). In this study, S. iron concentration and TSAT significantly decreased as early as 6 h after OHE, while TIBC decreased 18 to 32 h after surgery, and UIBC significantly increased 6 h after surgery in both the control and HBOT groups. Inflammation-related hypoferremia is multi-factorial. Upregulation of acute phase proteins such as ferritin and hepcidin leads to the sequestration of iron by the sarcoplasmic reticulum and the reduction of dietary iron absorption, resulting in increased UIBC (41–45). Simultaneously, the downregulation of the main iron binding transporter protein, transferrin, decreases TIBC (46). As the blood level of iron continues to decline, the amount of transferrin saturated with iron decreases, reflected by a lower TSAT.

To the author's knowledge, there are no published reports on the effects of HBOT on iron homeostasis. Iron and oxygen homeostasis are known to be strongly interconnected. Hypoxia is associated with decreased production of hepcidin and increased expression of transferrin. This results in an increase in dietary iron absorption and iron transport to erythroid tissues, ultimately leading to an increase in the capacity of red blood cells to transport oxygen (47). In the present study, the hyperoxemic state induced by HBOT did not have a detectable effect on iron homeostasis 32 h after surgery. Further studies are warranted to evaluate the effect of HBOT on the expression of hepcidin, transferrin and ferroportin in patients with systemic inflammation and/or ischemic injury.

Ovariohysterectomy induced a significant increase in KC-like and IL-6 serum concentration 6 h after surgery, with no significant change in the remaining cytokines and chemokines measured. Cytokines are key modulators of the inflammatory response to trauma and proper healing. Under physiologic conditions, a dynamic balance between pro- and anti-inflammatory cytokines serve as immunomodulatory elements to limit potential injury or excessive inflammatory reactions (48). Studies in human medicine yield insights into potential applications of cytokines in the diagnosis, monitoring, prognostication, and therapy of a variety of diseases (48), but much remains to be elucidated regarding cytokine regulation, synergistic or antagonistic interactions, and roles in either development, progression, or control of pathologic conditions in veterinary medicine. Previous studies report an increase in serum concentrations of IL-6 and IL-10 in healthy dogs 3 days after ovariohysterectomy (49) and identified KC-like as being significantly higher in dogs with pyometra and sepsis compared to dogs without sepsis (50). Additionally, IL-6 and KC-like have been shown to correlate with the severity of disease and surgical trauma (51, 52). The temporary and mild increase limited to IL-6 and KC-like identified in our study suggests that the inflammation induced by OHE was minimal. The absence of significant change between the control and HBOT groups suggests that HBOT does not induce alterations in pro- and anti-inflammatory cytokines in dogs after OHE. These findings are in contrast with previous studies that report an anti-inflammatory effect of HBOT in people and rodents treated for pancreatitis, traumatic brain injury, sepsis, and acute lung injury (4, 5, 13, 29, 53). In this study, the absence of detectable changes in cytokines levels after HBOT may be related to the nature of the surgery that induced a relatively minimal inflammatory response, and/or the inclusion of cytokines that are not specifically stimulated by OHE in dogs. Additionally, given the high inter-individual variability in the cytokine concentrations and response to stimulus, the small sample size may be a limitation to detect significant changes after OHE and HBOT. As previously mentioned, further studies evaluating the effects of HBOT on chronic and/or more severe inflammation are warranted in dogs to complement our investigation.

It is well-accepted that HBOT increases the production of reactive oxygen species at a sub-lethal concentration (12). At a low dose, via non-cytotoxic oxidative stimuli, increased reactive oxygen species levels activate a negative feedback loop, which leads to downregulation of oxidant enzymes and upregulation of antioxidant enzymes (54–57). One of the primary goals of this study was to determine the effects of HBOT on oxidative stress in dogs after OHE. For this purpose, TAS and TOS were measured to estimate the global antioxidant and oxidant status, respectively. To demonstrate the antioxidant effect of HBOT, most studies used individual antioxidant enzymes such as superoxide dismutase-1, catalase, and glutathione peroxidase, or oxidant enzymes such as malondialdehyde and F2-isoprostane (3–5, 58, 59). However, because the antioxidant and oxidant effects of each individual enzymes are additive, and their measurement can be technically challenging, none of these are ideal surrogate biomarker of oxidative stress (60). More recently, TAS and TOS have been suggested to assess the antioxidant and oxidant status of biological samples (24, 25, 61). In 2014, Lee et al. demonstrated an increase in plasma TOS and OSI levels and decrease in TAS levels after ovariectomy in dogs, suggesting the oxidative stress induced by the surgery (62). Total antioxidant and oxidant status were also used to demonstrate the antioxidant effects of HBOT in rats with induced acute lung injury after 5 daily treatments at 2.0 ATA for 1 h (53). In this study, OHE and HBOT did not induce any change in TOS, TAS, or OSI. These results may be related to the absence of oxidative stress induced by OHE performed by a board-certified surgeon and/or the lack of antioxidant effect of HBOT in dogs with no oxidative stress. A transient change in oxidation status after OHE or HBOT may be detected with collection of blood samples immediately after the procedures. The technique used to measure the total antioxidant and oxidant capacity may also be a source of error. To this point, the ideal reference method to measure TAS and TOS in dogs has not been established and further studies are needed to validate the technique used in this study (61). Finally, TAS and TOS estimate the antioxidant and oxidant capacity of the serum but provide limited information regarding the intracellular oxidative stress, and do not evaluate the role of important enzymes such as superoxide dismutase, glutathione peroxidase, and catalases (63, 64). Ideally, individual antioxidants and oxidants can be measured in complement to provide a wider picture of the antioxidant status.

A considerable number of studies suggests that short exposure to hyperoxia during HBOT can have therapeutic benefits. However, oxygen, particularly at supraphysiological pressures, also has the potential to increase systemic oxidative stress and induce hyperoxic cellular damage (65). Studies have shown that long-term exposure to HBOT (66, 67), and hyperbaric treatments at more than 3.0 ATA (29) results in cumulative oxidative stress and worsens clinical outcome in people and laboratory animals. This study identified a safe HBOT protocol for healthy dogs undergoing an elective surgical procedure.

In this study, HBOT did not improve wound healing or pain scores. The absence of effects on incision score after OHE is consistent with one study in which HBOT did not influence the healing of uncomplicated open and incisional wounds surgically created in dogs (68). There was no difference at any time for contraction, epithelialization, subjective wound scores, histopathology scores, or bacterial loads between the control group and the group that received HBOT (68). A Cochrane review including 12 randomized trials (577 human patients) concluded that HBOT is more likely to benefit ischemic wounds, or chronic wounds characterized by hypoxic tissues and associated with venous diseases or diabetes (69). Numerous studies have demonstrated an antinociceptive effect of HBOT for relief of chronic pain (70–72) and acute inflammatory pain in people (73, 74). The mechanism of the reported analgesic effect of HBOT is unknown, although some have attributed this effect to its anti-inflammatory properties and decreased expressions of inducible nitric oxide synthase and neuronal nitric oxide synthase (72, 73). In this study, there was no difference in pain scores or need for rescue analgesia between the control and HBOT groups. This is likely related to appropriate analgesia provided by the administration of opioids.

## Conclusion

This study identified a well-tolerated HBOT protocol with no adverse effects detected in dogs following elective surgery with minimal inflammation. The absence of significant changes in circulating inflammatory markers suggests that there are no added benefits of this HBOT protocol in elective procedure such as OHE. However, these findings do not apply to more severe or chronic diseases processes. Additional investigation into various sources and types of inflammation is required to identify appropriate markers to quantify the response to HBOT, determine its role as an adjunctive therapy in dogs, and optimal dose. The present study, however, provides an important prospective study on the safety and efficacy of HBOT.

## Data Availability Statement

All datasets generated for this study are included in the article/supplementary material.

## Ethics Statement

The animal study was reviewed and approved by Auburn University Institutional Animal Care and Use Committee.

## Author Contributions

AG, EG, LB, AW, LP, and KK contributed to the conception and design of the study. AG and KK supervised the hyperbaric oxygen treatments. AG, EG, and EF collected the data, contributed to the laboratory work, and analyzed the results. AG wrote the first draft of the manuscript. AG, EG, LB, EF, AW, LP, and KK contributed to the manuscript revision, read and approved the submitted version.

### Conflict of Interest

AG and KK received a research grant from Hyperbaric Veterinary Medicine®, Boca Raton, FL, USA. The funder was not involved in the study design, collection, analysis, interpretation of data, the writing of this article or the decision to submit it for publication. The remaining authors declare that the research was conducted in the absence of any commercial or financial relationships that could be construed as a potential conflict of interest.

## Abbreviations

ATA: atmosphere of absolute pressure
CRP: C-reactive protein
GM-CSF: granulocyte-macrophage colony-stimulating factor
HBOT: hyperbaric oxygen therapy
IL: interleukin
IFN-γ: interferon γ
IP-10: interferon-γ-induced protein
KC-like: keratinocyte chemotactic-like
MCP-1: monocyte chemoattractant protein 1
OHE: ovariohysterectomy
OSI: oxidative stress index
S. iron: serum iron
TAS: total antioxidant status
TIBC: total iron binding capacity
TOS: total oxidant status
TNF-α: tumor necrosis factor α
TSAT: transferrin saturation
UIBC: unsaturated iron-binding capacity.
